# Kinship analysis of *mecA* gene of methicillin-resistant *Staphylococcus aureus* isolated from milk and risk factors from the farmers in Blitar, Indonesia

**DOI:** 10.14202/vetworld.2024.216-225

**Published:** 2024-01-25

**Authors:** Aswin Rafif Khairullah, Shendy Canadya Kurniawan, Sri Agus Sudjarwo, Mustofa Helmi Effendi, Agus Widodo, Ikechukwu Benjamin Moses, Abdullah Hasib, Reichan Lisa Az Zahra, Maria Aega Gelolodo, Dyah Ayu Kurniawati, Katty Hendriana Priscilia Riwu, Otto Sahat Martua Silaen, Daniah Ashri Afnani, Sancaka Cashyer Ramandinianto

**Affiliations:** 1 Division of Animal Husbandry, Faculty of Veterinary Medicine, Universitas Airlangga, Jl. Dr. Ir. H. Soekarno, Kampus C Mulyorejo, Surabaya 60115, East Java, Indonesia; 2 Master Program of Animal Sciences, Department of Animal Sciences, Specialisation in Molecule, Cell and Organ Functioning, Wageningen University and Research, Wageningen 6708 PB, Netherlands; 3 Division of Basic Veterinary Medicine, Faculty of Veterinary Medicine, Universitas Airlangga, Jl. Dr. Ir. H. Soekarno, Kampus C Mulyorejo, Surabaya 60115, East Java, Indonesia; 4 Division of Veterinary Public Health, Faculty of Veterinary Medicine, Universitas Airlangga, Jl. Dr. Ir. H. Soekarno, Kampus C Mulyorejo, Surabaya 60115, East Java, Indonesia; 5 Department of Health, Faculty of Vocational Studies, Universitas Airlangga, Jl. Dharmawangsa Dalam Selatan No. 28-30, Kampus B Airlangga, Surabaya 60115, East Java, Indonesia; 6 Department of Applied Microbiology, Faculty of Science, Ebonyi State University, Abakaliki 480211, Nigeria; 7 School of Agriculture and Food Sustainability, The University of Queensland, Gatton, QLD 4343, Queensland, Australia; 8 Profession Program of Veterinary Medicine, Faculty of Veterinary Medicine, Universitas Airlangga, Jl. Dr. Ir. H. Soekarno, Kampus C Mulyorejo, Surabaya 60115, East Java, Indonesia; 9 Department of Animal Infectious Diseases and Veterinary Public Health, Faculty of Medicine and Veterinary Medicine, Universitas Nusa Cendana, Jl. Adisucipto Penfui, Kupang 85001, East Nusa Tenggara, Indonesia; 10 Indonesia Research Center for Veterinary Science, Jl. RE Martadinata No. 30, Bogor 16114, West Java, Indonesia; 11 Department of Veterinary Public Health, Faculty of Veterinary Medicine, Universitas Pendidikan Mandalika, Jl. Pemuda No. 59A, Dasan Agung Baru, Mataram 83125, West Nusa Tenggara, Indonesia; 12 Doctoral Program of Biomedical Science, Faculty of Medicine, Universitas Indonesia, Jl. Salemba Raya No. 6 Senen, Jakarta 10430, Indonesia; 13 Department of Microbiology and Parasitology, Faculty of Veterinary Medicine, Universitas Pendidikan Mandalika, Jl. Pemuda No. 59A, Dasan Agung Baru, Mataram 83125, West Nusa Tenggara, Indonesia; 14 Lingkar Satwa Animal Care Clinic, Jl. Sumatera No. 31L, Gubeng, Surabaya 60281, East Java, Indonesia

**Keywords:** hand swab, *mecA*, methicillin-resistant *Staphylococcus aureus*, milk, public health

## Abstract

**Background and Aim:**

There are numerous reports of subclinical mastitis cases in Blitar, which is consistent with the region’s high milk production and dairy cattle population. *Staphylococcus aureus*, which is often the cause of mastitis cases, is widely known because of its multidrug-resistant properties and resistance to β-lactam antibiotic class, especially the methicillin-resistant *S. aureus* (MRSA) strains. This study aimed to molecular detection and sequence analysis of the *mecA* gene in milk and farmer’s hand swabs to show that dairy cattle are reservoirs of MRSA strains.

**Materials and Methods:**

A total of 113 milk samples and 39 farmers’ hand swab samples were collected from a dairy farm for the isolation of *S. aureus* using Mannitol salt agar. The recovered isolates were further characterized using standard microbiological techniques. Isolates confirmed as *S. aureus* were tested for sensitivity to antibiotics. Oxacillin Resistance Screening Agar Base testing was used to confirm the presence of MRSA, whereas the *mecA* gene was detected by polymerase chain reaction and sequencing.

**Results:**

A total of 101 samples were confirmed to be *S. aureus*. There were 2 *S. aureus* isolates that were multidrug-resistant and 14 *S. aureus* isolates that were MRSA. The *mecA* gene was detected in 4/14 (28.6%) phenotypically identified MRSA isolates. Kinship analysis showed identical results between *mecA* from milk and farmers’ hand swabs. No visible nucleotide variation was observed in the two *mecA* sequences of isolates from Blitar, East Java.

**Conclusion:**

The spread of MRSA is a serious problem because the risk of zoonotic transmission can occur not only to people who are close to livestock in the workplace, such as dairy farm workers but also to the wider community through the food chain.

## Introduction

An infectious disease known as “food-borne disease” is caused by consuming water and other foods contaminated with pathogenic organisms, poisons, and chemicals [1]. Bacteria (66%), parasites (4%), viruses (4%), and chemicals (26%) are the primary causes of food-borne illness [2]. Cow’s milk is an animal-derived dietary ingredient that has the potential to spread several pathogenic germs that could have an effect on public health, often known as milk-borne illness [3].

This is because milk is a nutrient-rich substrate appropriate for the growth and spread of harmful germs [4], which includes chemical components that the body needs. The primary ingredients of milk are minerals (0.7%), fat (3.7%), protein (3.5%), lactose (4.9%), and water (87.2%) [5]. Dairy products should have a pH between 6.5 and 6.6 to be most hospitable to microorganisms because a pH between 6.5 and 7.5 is ideal for bacterial growth and rapid deterioration [6].

Milk contaminated with pathogenic bacteria (milk-borne pathogens) can easily be contaminated anytime and anywhere if handled improperly [7]. The high level of contamination during the milking process may be due to the large spread of pathogenic microorganisms [8]. Among the risk factors for bacterial contamination are farmers’ dirty hands during milking, dairy equipment’s lack of sterility, the environment around the cowsheds, the proximity of manure disposal sites to the cowsheds, and the proximity of the cowsheds to wells [9]. *Staphylococcus aureus* is one of the most common pathogenic bacteria contaminating milk [10].

The opportunistic pathogen *S. aureus* can cause several infectious illnesses in humans and animals [11]. This bacterium can spread in the air, animals, humans, and contaminated surfaces [12]. According to reports, *S. aureus* is frequently found in the raw milk of both healthy animals and those with subclinical mastitis [13]. *S. aureus* remains the most common pathogenic bacteria isolated from mastitis milk samples [14]. However,

Blitar Regency, East Java province is one of the regions where Indonesia’s largest fresh cow’s milk is produced [15]. In line with the high amount of milk production and the dairy cattle population in Blitar, reports of cases of subclinical mastitis are also found with the habits of breeders who pay little attention to the care of dairy cows affected by subclinical mastitis, which will eventually develop into clinical mastitis [16]. The management of cases of mastitis is often achieved by administering antibiotics [17]. Several antibiotics, including oxytetracycline, penicillin, and ampicillin, are often used to treat mastitis; however, based on information from animal health workers in Blitar [18], these antibiotics are no longer effective for treating mastitis in dairy cows.

*S. aureus*, which is often the cause of mastitis cases, is widely known because of its multidrug-resistant (MDR) properties and its resistance to β-lactam class antibiotics, particularly methicillin-resistant *S. aureus* (MRSA) strains [19]. MRSA has been implicated in a series of nosocomial infections [20]. All β-lactam medications, such as cephalosporins and carbapenems, are considered ineffective against MRSA strains resistant to oxacillin (OX) and cefoxitin (FOX) [21]. It has been noted that gentamicin, ciprofloxacin, and clindamycin resistance in animal MRSA isolates is much higher than that in human MRSA isolates [22].

The *mecA* gene mediates resistance to beta-lactam antibiotics in MRSA strains [23]. This gene is found on the staphylococcal cassette chromosome *mec* (SCC*mec*), a cellular genetic component [24]. This gene produces penicillin-binding protein 2a (PBP 2a), which has a lower affinity for β-lactam antibiotics [25]. Methicillin-resistant *S. aureus* containing the *mecA* gene will be resistant to β-lactam class antibiotics [26].

The incidence of MRSA infection can be a public health problem; therefore, laboratory tests, molecular detection, and sequence analysis of the *mecA* gene are needed to prove that cows are reservoirs for the emergence of the MRSA strain. In view of the certainty of contamination of food reservoirs by *mecA*-harboring MRSA, it will be easy to overcome the occurrence of MRSA infection in foods of animal origin, especially milk.

This study aimed to molecular detection and sequence analysis of the *mec*A gene in milk and farmer’s hand swabs to show that dairy cattle are reservoirs of MRSA strains.

## Materials and Methods

### Ethical approval

This study was approved by the Health Research Ethical Clearance Commission, Universitas Airlangga (No. 353/HRECC.FODM/VI/2021). All the methods used in this study were performed in accordance with relevant guidelines and regulations.

### Study period and location

This study was conducted from March to June 2022 at Faculty of Veterinary Medicine, Universitas Airlangga.

### Sample collection and the study area

A total of 113 milk samples and 39 farmers’ hand swab samples were used in this study. Milk sampling was performed on lactating cows while hand swab sampling was performed on the palms of farmers after milking in Blitar Regency, East Java, Indonesia. Collected samples were labeled and immediately transported to the laboratory in a cool box containing an ice-pack gel. Cow milk samples and hand swabs from farmers were collected from several farms in Blitar Regency, East Java, Indonesia. Sample examination was carried out at the Veterinary Public Health Laboratory, Faculty of Veterinary Medicine, Airlangga University.

### Isolation and identification

First, a milk sample and farmer’s hand swab were taken using a loop, then spread in a zigzag manner on NA media (Oxoid, UK) and incubated for 24 h at 37°C. Bacterial isolates grown on nutrient agar media were collected using a loop, streaked in a zigzag manner on mannitol salt agar (MSA) media (Oxoid), and incubated for 24 h at 37°C [27]. Suspected *S. aureus* colonies (golden-yellow) were further characterized using standard microbiology techniques, such as Gram staining, coagulase, catalase, β-galactosidase, pyrrolidonyl arylamidase, and acetoin production tests [28].

### Antibiotic susceptibility test

We used the Kirby-Bauer disk diffusion method in accordance with the guidelines of the Clinical and Laboratory Standards Institute (CLSI) [29]. Identified *S. aureus* isolates were first standardized to a McFarland turbidity of 0.5 in test tubes. Sterile cotton swabs were then dipped into each standardized inoculum, drained to remove excess inoculum, and uniformly inoculated on Mueller-Hinton agar (MHA) (Oxoid), and allowed to dry for a few minutes. Antibiotic-impregnated disks: Gentamicin (10 μg), erythromycin (E) (15 μg), tetracycline (TE) (30 μg), FOX (30 μg), and OX (30 μg) – for MRSA screening and MRSA screening, respectively. Each antibiotic disk on the MHA plate was separated by at least 25–30 mm. Inhibition zone diameter was measured, recorded, and the results were interpreted as susceptible or resistant based on CLSI guidelines [29].

### Confirmatory MRSA test results

Identified MRSA isolates (resistance to FOX and OX) from the antimicrobial susceptibility test results were further confirmed by streaking them on Oxacillin Resistance Screening Agar Base (ORSAB) (Himedia, India) before incubation for 24 h at 37°C [30]. The appearance of blue colonies after 24 h of incubation is indicative of MRSA.

### Genotype detection

DNA was extracted using a QIAamp DNA Mini Kit (51304 and 51306) (Hildenberg, Germany). Specific primers for the *mecA* gene were used as described in Table-1 [26]. Polymerase chain reaction (PCR) mixture of 18.75 μL consisting of 12.5 μL PCR Mastermix (0.2 mM dNTP, 0.5 U Taq polymerase, buffer, and 1.5 mM MgCl_2_), 1.25 μL for each primer, and 5 μL DNA template was prepared. The PCR reaction was conducted under the following conditions: initial denaturation at 94°C for 7 min, 35 cycles of 96°C for 50 s of denaturation, 50°C for 40 s of annealing, 72°C for 1 min of extension, and finally, 10 min of extension at 72°C [26]. A total volume of 5 μL of the amplified PCR products was analyzed by 1.5% agarose gel electrophoresis and visualized in an ultraviolet transilluminator (Cole-Parmer, UK) with a wavelength of 360 nm [31].

**Table-1:** Details of primers used in this study.

Primers	Sequences (5’–3’)	Target gene	Amplicons size	Reference
*mecA* forward	5’- AAA ATC GAT GGT AAA GGT TGG C-3’	*mecA*	533 bp	[26]
*mecA* reverse	5’- AGT TCT GCA GTA CCG GAT TTG C-3’	*mecA*		

### Sequencing

Amplified PCR products positive for *mecA* were purified in a volume of 20 μL and sequenced to determine the nucleotide base sequence of the *mecA* genes. PCR sequencing was followed by labeling using pure DNA, one of the primers, a terminator, buffer, and distilled water. Sequencing was performed using the Applied Biosystem tool (Warrington, UK). Sequencing results are presented in the form of a chromatogram graph with different colors for each nitrogen base in the DNA. On the basis of the obtained sequencing results, homology, phylogenetics, mutations, evolution, and protein characteristics can be determined. The obtained sequencing results were then analyzed at National Center for Biotechnology Information (NCBI) (Maryland, USA) [32].

Molecular Evolutionary Genetics Analysis version X (https://www.megasoftware.net/) was used to assess the sequencing results. Reference *mecA* sequences were obtained from GenBank (NCBI) and selected based on species after Basic Local Alignment Search Tool (BLAST) analysis of *mecA* sequences of isolates in constructing a phylogenetic tree [33].

## Results

### Isolation and identification methods

**Figure-1:**
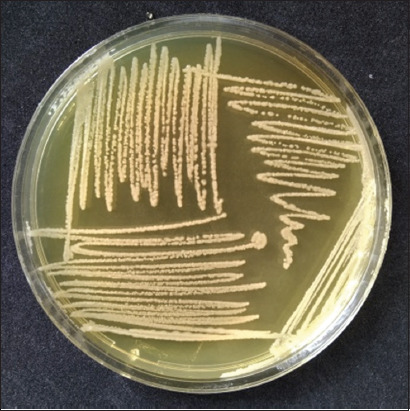
Golden-yellow colonies of *Staphylococcus aureus* on mannitol salt agar.

Of the 152 samples collected, 101 (66.48%) were positive for *S. aureus* (Table-2 and Figure-1).

**Table-2:** Isolation and identification of *S. aureus.*

Type of sample	Sample size	Isolation test on MSA	Identification test			Positive *S. aureus* (%)

			Gram stain	Biochemical test		

				Catalase	Coagulase	
Milk	113	113	113	113	73	73 (64.6)
Hand swab	39	39	39	39	28	28 (71.8)
Total						101 (66.48)

*S. aureus*=*Staphylococcus aureus*, MSA=Mannitol salt agar

### Antibiotic sensitivity test

*S. aureus* strains were resistant to TE (44), OX (31), E (10), FOX (14), and gentamicin (2) (Table-3).

**Table-3:** Isolated *S. aureus* resistance profile by antibiotic group.

Group of antibiotics	Resistance profile	Number of isolates (n=101)	Total number of isolates (%)

		Resistant isolates (%)	
0	No one is resistant	36 (35.64)	36 (35.64)
1	OX	8 (7.92)	45 (44.55)
	TE	26 (25.74)	
	E	1 (0.99)	
	GM	2 (1.98)	
	FOX–OX	8 (7.92)	
2	OX–TE	8 (7.92)	18 (17.82)
	OX–E	1 (0.99)	
	TE–E	5 (4.95)	
	OX–FOX–TE	3 (2.97)	
	OX–FOX–E	1 (0.99)	
≥3	OX–FOX–TE–E	2 (1.98)	2 (1.98)

GM=Gentamicin, E=Erythromycin, FOX=Cefoxitin, TE=Tetracycline, OX=Oxacillin, *S. aureus*=*Staphylococcus aureus*

**Figure-2:**
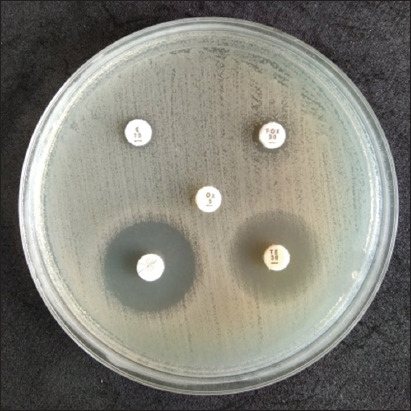
Antibiotic susceptibility test conducted on *Staphylococcus aureus* on Mueller-Hinton agar.

Of 101 recovered *S. aureus* isolates, 36 isolates were not resistant to all antibiotics tested, 45 isolates (44.55%) were resistant to 1 class of antibiotics tested, 18 isolates (17.82%) were resistant to 2 classes of antibiotics, and 2 isolates (1.98%) were confirmed to be MDR because they were resistant to 3 classes of antibiotics (Figure-2) with a pattern of antibiotic resistance OX–FOX–TE–E (Table-4). This could explain the low frequency of *S. aureus* MDR cases reported in Blitar Regency because only two MDR isolates were recovered from 152 analyzed samples.

**Table-4:** *S. aureus* isolates with a profile MDR.

Location	Sample code	Resistant group	Antibiotic				

			OX	FOX	TE	E	GM
Milk	SS 9	OX–FOX–TE–E	✓	✓	✓	✓	**–**
Hand swab	ST 29	OX–FOX–TE–E	✓	✓	✓	✓	**–**

✓=Resistant, GM=Gentamicin, E=Erythromycin, FOX=Cefoxitin, TE=Tetracycline, OX=Oxacillin, MDR=Multidrug resident, *S. aureus*=*Staphylococcus aureus*

### Confirmatory MRSA test results

**Figure-3:**
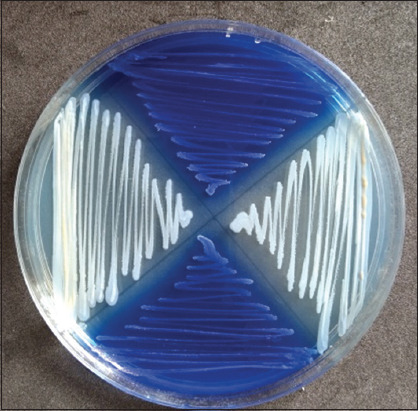
Oxacillin resistance screening agar base test for methicillin-resistant *Staphylococcus aureus* (MRSA) identification, blue colonies indicate MRSA positive.

The ORSAB test revealed that 14 out of 31 *S. aureus* isolates that were resistant to FOX and OX were positive for MRSA (Table-5 and Figure-3. It shows that the level of MRSA infection in dairy farms in Blitar Regency is still low, as only 14 MRSA isolates were recovered from the 152 analyzed samples.

**Table-5:** Total number confirmed MRSA by ORSAB.

Sample type	Resistant group of antibiotics	Number of isolates tested by ORSAB (n=31)	Positive ORSAB test (%)	Number of MRSA (%)
Milk	OX	5	1 (3.22)	11 (35.48)
	OX–FOX	6	5 (16.13)	
	OX–TE	6	1 (3.22)	
	OX–E	1	0 (0)	
	OX–FOX–TE	3	2 (6.45)	
	OX–FOX–E	1	1 (3.22)	
	OX–FOX–TE–E	1	1 (3.22)	
Hand swab	OX	3	0 (0)	3 (9.68)
	OX–FOX	2	2 (6.45)	
	OX–TE	2	0 (0)	
	OX–FOX–TE–E	1	1 (3.22)	
Total		31	14 (45.16)	14 (45.16)

*S. aureus* isolates screened for ORSAB test were only *S. aureus* isolates that were resistant to b-lactam antibiotics (cefoxitin and oxacillin). E=Erythromycin, FOX=Cefoxitin, TE=Tetracycline, OX=Oxacillin, MRSA=Methicillin-resistant *Staphylococcus aureus*, ORSAB=Oxacillin Resistance Screening Agar Base

### Genotype detection

**Figure-4:**
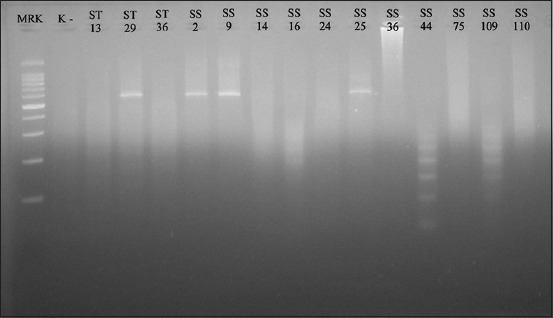
Positive bands at 533 bp in the detection *mecA* polymerase chain reaction findings; marker line: Molecular weight indicators of 100 bp; Line K-: Negative control; Line ST29, SS2, SS9, and SS25: *mecA* gene-positive isolate.

Four phenotypically identified MRSA isolates carried the *mecA* gene based on the electrophoresis results of the 14 isolates investigated. Three of these isolates harboring the *mecA* genes were recovered from milk, whereas one (1) was recovered from a farmer’s hand swab (Table-6 and Figure-4).

**Table-6:** *mecA* gene detection based on ORSAB test.

Sampel type	Sample code	Resistant group of antibiotics	ORSAB test (n=31)	*mecA* detection	Number positive of MRSA isolates by *mecA* detection (%)
Milk	SS 1	OX	–	Not tested	3 (9.68)
	SS 2	OX–FOX–TE	+	+	
	SS 9	OX–FOX–TE–E	+	+	
	SS 13	OX–TE	–	Not tested	
	SS 14	OX–FOX	+	–	
	SS 15	OX	–	Not tested	
	SS 16	OX–FOX	+	–	
	SS 21	OX–FOX–TE	–	Not tested	
	SS 24	OX–FOX	+	–	
	SS 25	OX	+	+	
	SS 30	OX–FOX–E	–	Not tested	
	SS 36	OX–TE	+	–	
	SS 37	OX–TE	–	Not tested	
	SS 38	OX–TE	–	Not tested	
	SS 44	OX–E	+	–	
	SS 52	OX–FOX	–	Not tested	
	SS 53	OX–FOX–TE	–	Not tested	
	SS 75	OX	+	–	
	SS 90	OX	–	Not tested	
	SS 98	OX–TE	–	Not tested	
	SS 100	OX –TE	–	Not tested	
	SS 109	OX–FOX	+	–	
	SS 110	OX–FOX	+	–	
Hand swab	ST 10	OX	–	Not tested	1 (3.22)
	ST 13	OX–FOX	+	–	
	ST 17	OX–TE	–	Not tested	
	ST 21	OX	–	Not tested	
	ST 24	OX	–	Not tested	
	ST 27	OX–TE	–	Not tested	
	ST 29	OX–FOX–TE–E	+	+	
	ST 36	OX–FOX	+	–	
Total					4 (12.9)

*mecA* gene detection was only carried out on *S. aureus* isolates that were confirmed positive on the ORSAB test. E=Erythromycin, FOX=Cefoxitin, TE=Tetracycline, OX=Oxacillin, ORSAB=Oxacillin Resistance Screening Agar Base

### Sequencing

**Figure-5:**
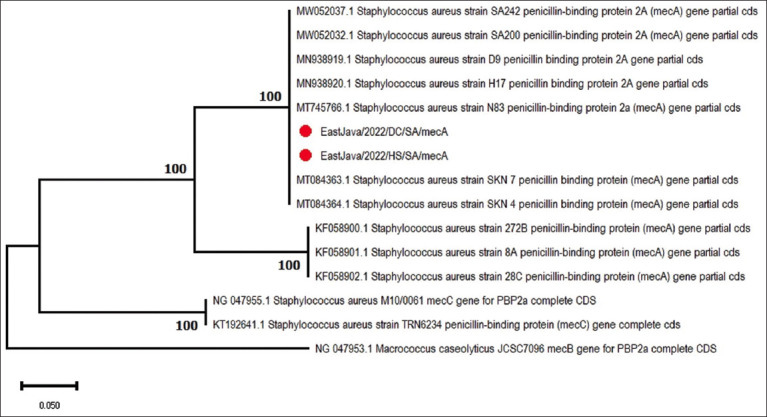
Maximum likelihood phylogenetic tree from partial sequencing of the *Staphylococcus aureus* *mecA* gene.

Of the four MRSA isolates harboring the *mecA* gene, two isolates were selected (one isolate from a milk sample and one isolate from a farmer’s hand) for sequencing to perform kinship analysis. The genetic relationship analysis showed that EastJava/2022/HS/SA/*mecA* and EastJava/2022/DC/SA/*mecA* clustered together with seven sequences of *S. aureus* PBP 2a (*mecA*) genes from India, Iran, and Egypt (Figure-5).

## Discussion

This study identified MDR *S. aureus* isolates and MRSA as contaminant bacterial pathogens in dairy farms in Blitar Regency, East Java, Indonesia, isolated from cow milk and farmers’ hands. *MecA*-harboring MRSA was also isolated.

The highest frequency of TE resistance among the *S. aureus* isolates in this study was found in 44 (43.56%) out of the 101 isolates recovered. TE inhibits protein synthesis by binding to the 30S ribosomal subunit [34]. The mechanism of its inhibitory action involves preventing aminoacyl-tRNA from binding to the acceptor site on the mRNA-ribosome complex, which prevents amino acids from being incorporated into the peptide chain [31]. This interference also prevents the tRNA-carrying amino acids from attaching to the 30S or 70S ribosomes, thus preventing the amino acids from being added to the peptide chain as it grows [35]. Two well-known mechanisms of TE resistance are active drug efflux from the *tetK* gene and ribosomal protection through competitive binding of the 30S subunit by ribosomal protection protein, which is generated by the *tetM* gene [36].

The frequency of resistance of *S. aureus* to gentamicin was the lowest as only two out of 101 isolates were resistant to this aminoglycoside. Gentamicin expresses its activity against bacteria by attaching to the 16S ribosomal RNA aminoacyl site of the 30S ribosomal subunit, preventing or inhibiting the translation of the genetic code [37]. As noted in this study, the low resistance frequency of S. aureus isolates to gentamicin might be due to minimal exposure to aminoglycoside class antibiotics in dairy farms in our study area. Interestingly, gentamicin could serve as an effective alternative treatment because of this low resistance. However, the reports of gentamicin-resistant strains in our study indicate that it is imperative to re-examine the risk factors for their transmission and the mechanisms of resistance [38].

Of the 101 *S. aureus* isolates recovered in this study, 2 (1.98%) were MDR as they exhibited resistance to at least three different antibiotic classes. The emergence of MDR bacteria occurs due to multistep mutation resulting in a gradual increase in resistance, where the majority of bacterial information is encoded by chromosomes [39]. Some bacteria carry extrachromosomal genes found in plasmids or bacteriophages [40]. Transposons and integrons are sites where transmitting plasmids, also known as factor R plasmids, transfer resistance factors from chromosomes to plasmids [41]. An integron is made up of two DNA segments with an antibiotic resistance gene on one side, as opposed to a transposon, which is one gene or a small number of resistance genes that are directly repeated or reverse bound [42].

A total of 14 MRSA isolates were found on dairy farms in Blitar. SCCmec, a sizable DNA fragment between 20 and 100 kb, helps *S. aureus* to evolve into a methicillin-resistant strain (MRSA) [43]. SCC*mec* integrates into the *S. aureus* chromosome in a region near the origin of chromosomal replication [44]. The normal PBP, specifically PBP 2 to PBP 2a, has undergone modifications that have led to MRSA isolates being resistant to all β-lactam class drugs [45]. PBP 2a has a very low affinity for β-lactams; therefore, even when this bacterium is cultivated in media with high concentrations of β-lactams, the MRSA strain may still survive and construct the bacterial cell wall [46].

MRSA is a pathogenic bacterial strain that is typically found in humans but can also colonize and infect other animals, including livestock, wildlife, pets, and poultry [47]. MRSA infection in animals is important from an animal welfare and economic perspective and can also act as a reservoir for human zoonotic infections [48]. MRSA infection is difficult to treat because it is resistant to various antibiotics and spreads easily. Therefore, early detection of MRSA infection is very necessary [49].

In this study, four MRSA isolates were shown to contain the *mecA* gene. *S. aureus* with MRSA characteristics is a strain of pathogenic bacteria encoded by several resistance genes, one of which is the *mecA* gene [50]. In 2007, the *mecA* gene was first isolated from an MRSA isolate originating from milk of dairy cows in England [51]. The *mecA* gene of MRSA bacteria is a determining factor in the occurrence of antibiotic resistance [52]. This type of antibiotic resistance occurs in the β-lactam antibiotic class, where resistance occurs due to the presence of PBP2a protein [53].

The SCC*mec* chromosome of MRSA contains the *mecA* gene, which encodes a specific transpeptidase that makes the bacteria resistant to methicillin and β-lactam drugs [54]. This gene produces PBP 2a [55]. Antibiotics belonging to the β-lactam class have a low affinity for this protein [56]. Bacteria that generate this protein will be able to withstand all types of β-lactam class antibiotics [57]. Therefore, the resistance of MRSA bacteria is encoded by the *mecA* gene [58].

With the discovery of MRSA isolates carrying the *mecA* gene in the milk of dairy cows and farmer’s hand swabs in our study, it is necessary to conduct a comprehensive evaluation of milking hygiene, handling of milk and dairy products, judicious use of antibiotics, and sanitation management to control the transmission of MRSA between farmers and dairy cows and the health of consumers, breeders, veterinarians, and the surrounding community [23].

The results of the phylogenetic analysis confirmed that two S. *aureus* isolates were obtained from milk and hand swabs of farmers at dairy farms in Blitar Regency, East Java. Sequencing results between the origin sequences of the cow’s milk sample (EastJava/2022/DC/SA/*mecA*) and the farmer’s hand swab (EastJava/2022/HS/SA/*mecA*) showed identical results to one another as there were no visible nucleotide variations in both sequences of the isolates from Blitar, East Java, although the two isolates came from different host sources.

The results of the phylogenetic tree analysis showed that isolates from milking hands (EastJava/2022/HS/SA/*mecA*) and milk (EastJava/2022/DC/SA/*mecA*) were in one cluster with seven sequences of S. *aureus* PBP 2a (*mecA*) partial gene cds from India, Egypt, and Iran and three sequences of S. *aureus* PBP (*mecA*) partial gene cds from Brazil in different clusters. Seven isolates from the same cluster, with two isolates from East Java, were isolated from cow’s milk (India), dog and horse nasal swabs (Egypt), and human clinical samples (Iran). This cluster showed a large host diversity compared to the second cluster, which consisted of only three samples of milk from mastitis cows in Brazil.

The results of the three analyses indicated the potential for the two clusters in the phylogenetic tree to originate from two different MRSA strains. Follow-up tests and analyses with multilocus sequence typing (MLST) are required to determine the strains of the two isolates of cow’s milk and milking hands from East Java in this study. Spa typing is also recommended to differentiate the type of *S. aureus* [59].

In the context of One Health, MRSA is a serious problem because its risk of zoonotic transmission can occur not only to people who are close to livestock in the workplace, such as dairy farm workers but also to the wider community through the food chain [50]. There is increasing evidence showing that livestock is an important reservoir of MRSA strains that have the potential for zoonotic transmission [60]. Intraspecies and interspecies transmission of MRSA, including zoonotic transmission of MRSA strains from livestock to humans, has consistently been identified as having the highest risk of contracting MRSA among people living and working near livestock [61].

## Conclusion

This study has shown that cow milk and farmers’ hands in dairy farms located in Blitar Regency, East Java, Indonesia are potential reservoirs of MDR *S. aureus*. Clinically important *mecA* harboring MRSA were also detected. There was also a genetic link between the *mecA* gene derived from dairy cow milk and hand swabs obtained from farmers. A kinship analysis carried out to determine the genetic relatedness/diversity of the *mecA* genes harbored by the MRSA isolates recovered in this study with other publicly available *mecA* genomes on the NCBI database showed that they clustered together with seven other *mecA* gene sequences of *S. aureus* reported in India, Iran, and Egypt. Detection of MDR *S. aureus*, including methicillin-resistant strains, in cow milk and farmers’ hands is worrisome, as this could draw back some of the good gains of One Health. Therefore, it is of the utmost importance to ensure the appropriate use of antibiotics in the field of veterinary medicine to limit the increasing spread of antibiotic resistance.

## Authors’ Contributions

ARK and SCK: Conceived, designed, and coordinated the study. AW, AH, SCR and OSMS: Designed data collections tools, supervised the field sample and data collection, and laboratory work as well as data entry. KHPR, DAK, RLAZ, MAG and DAA: Contributed reagents, materials, and analysis tools. MHE, SAS, and IBM: Carried out the statistical analysis and interpretation and participated in the preparation of the manuscript. All authors have read, reviewed, and approved the final manuscript.
